# Pictorial support in health visits in Child Health Services provides or limits children’s space for participation

**DOI:** 10.1186/s12913-026-15049-1

**Published:** 2026-07-03

**Authors:** Marie Golsäter, Josefine Halldén, Jenny Lindholm Jansson, Maria Harder

**Affiliations:** 1 CHILD Research Group, School of Health and Welfare, Jönköping University, Jönköping, Sweden; 2 Child Health Services and Futurum – Academy for Health and Care, Region Jönköping County, Jönköping, Sweden; 3 Child Health Services, Region Jönköping County, Jönköping, Sweden; 4 Pediatric Outpatient Clinic, Region Jönköping County, Jönköping, Sweden; 5 School of Health, Care and Social Welfare, Mälardalen University, Västerås, Sweden

**Keywords:** Children, Child Health Services, Health visits, Participation, Pictorial support, Hermeneutic analysis

## Abstract

**Background:**

Children participate in different health care encounters throughout childhood and sufficient information and a sense of security are prerequisites for the child to participate on their own terms. Pictorial support is preferable for providing information and creating a common understanding among children, caregivers and parents. The aim of the present study is to explore five-year-olds’ experiences of using pictorial support during health visits in the Child Health Services from a participation perspective.

**Methods:**

This study used a qualitative design with a hermeneutic analysis. Seventeen five-year-old children were observed during ongoing health visits. The children were then interviewed about their experiences of using pictorial support.

**Results:**

Children’s experiences of using pictorial support from a participation perspective are conditioned by the nurses through *provision or limitation of space*, as their guidance may provide the children with *space to enable autonomy and a self-chosen pace* or *space to develop understanding*. The nurse’s guidance may also contribute to *limitation of space*,* which develops resignation*.

**Conclusion:**

In summary, children’s experiences of using pictorial support focusing on the child’s opportunity for participation seem to be conditional, affected by circumstances in the situation. It is not enough to provide space and let children use their voice through pictorial support if they have no audience willing to listen or opportunities to influence the visit.

**Supplementary Information:**

The online version contains supplementary material available at 10.1186/s12913-026-15049-1.

## Background

Children participate in different health care encounters throughout childhood and sufficient information and a sense of security are prerequisites for the child to participate on their own terms [1]. Furthermore, the information needs to be comprehensible for the child to understand what will happen [2, 3]. Nurses’ actions in the interaction with the children are essential. Such actions can, in a broader sense, mean that the nurses guide the children through the encounter by directive and flexible strategies to reach an agreement with the child to accomplish an examination [4]. In addition, the information must be designed based on the child’s needs, age and level of maturity [5, 6]. One way of designing information for children is using various forms of pictorial support. Pictorial support, such as photographs, drawings, or digital animations, can aid children in preparing themselves before and during a health care encounter. Pictorial support can facilitate children’s understanding of what will happen and thereby promote participation [7, 8]. Pictures and illustrations have been shown to help make the child more interested in the encounter [9] and support the child’s expression of thoughts [10].

Further, different kinds of pictorial support have been used to facilitate for children to express their needs and describe symptoms of, for example, nausea in conjunction with cancer treatment [11] and also regarding children undergoing needle-related procedures and pain management [8, 12].

The Swedish Child Health Services (CHS) aims to promote children’s (aged 0–5 years) development and health through regular health visits [13]. In the visits within CHS, the children are predominantly healthy children who undergo examinations of, e.g. language, hearing, and growth [14] as opposed to children who encounter health care as sick patients. This gives the child a better opportunity to participate on their own terms based on their well-being. Further, the examinations in CHS do not need to be carried out at the time in question if the child expresses fear or concern, as opposed to examinations and treatments at a hospital. As virtually every child takes part in these visits, the encounters constitute a base for their participation in health care situations both in the present and in future. In this study, James [15] description of participation is used: the child’s opportunity to take part in and contribute to a situation.

The intention of the Convention on the Rights of the Child states that every child has the right to receive understandable information adapted to their age, and understandable information is essential to promote participation [16]. When a child experiences trust, they are more likely to feel the prerequisite sense of security needed for participation during medical procedures [17]. In practice, this implies listening to the children to help them feel secure in a situation [9]. Health care professionals such as CHS nurses need to ensure that they promote the children’s participation in the encounter by adapt their clinical practice to the children’s ability [16]. Studies have shown how CHS nurses guide children through the various parts of a health visit and alternately use flexible and directed strategies to facilitate the children’s participation in the situation [4, 18]. When children visit health care, the parents are an essential part, and in child-centred care, the partnership is a triad; parent, child and health care staff, but the child is the key person in focus [19–20]. Pictorial support is preferable for providing information and creating a common understanding among children, caregivers and parents [21]. Further, such support aids children in understanding what will happen and having control in the situation; i.e., helping them feel secure and therefore promoting their participation [22].

To further develop understanding on how pictorial support can promote participation during health visits with young children in CHS, there is a need to explore children’s own views on using this type of support during such visits focusing on the child’s opportunity for participation.

## Aim

The aim of this study is to explore five-year-olds’ experiences of using pictorial support during health visits at the Child Health Services from a participation perspective.

## Methods

### Design and methodological approach

In this study a qualitativedesign with a hermeneutic analysis was adopted, and observations and interviews were used. A hermeneutic analysis entails that the researchers’ prior understanding is essential for the interpretive process and the movement between the children’s and researchers’ perspectives [23, 24]. Still, prior understanding is hard to describe, as it changes with the various experiences one acquires over time. The first author (MG) is a paediatric nurse and the last author (MH) a public health nurse, both with experience conducting research with children and nurses at the CHS, and working as development officers within the CHS. Furthermore, the researchers’ previous exploration and findings of children’s participation in various contexts have contributed to the hermeneutic analysis of this study as an essential part of the interpretive process [23].

### Study setting

The study was performed at one CHS clinic, and the population included children from urban and rural areas with varying socio-economic backgrounds in southeast Sweden. Seven CHS nurses work at the CHS clinic having their assignment to carry out the national CHS programme for about 2000 children i.e. promoting health and development among children. The national three-tier program is based on proportional universalism, which means that it is founded on all children having the opportunity to participate in a health-promoting and preventive program. From the outcome of the universal health visits, targeted interventions are designed based on the child’s and the family’s needs to achieve equal health for all children [13].

The universal health visit at five years of age involves measuring the child’s growth, dialogues about lifestyle habits and the child’s everyday functioning, and a vaccination. In addition, some children may also need an examination of, e.g., their vision or hearing. The CHS nurses carried out the visits according to the National CHS programme, meaning that no alterations were made due to the visits being observed.

### The pictorial support

A month before the visit a notice about the health visit is sent to the family by mail. The notice contains a reference to a website and a request to the parents to prepare the child before the visit by showing them a slide show available on the website. For each picture in the slide show, there is a short text for the child and a more elaborate text for the parents to facilitate for the parents to prepare the child. Each picture represents an examination to be performed during the visit and includes a description the parents can use in the dialogue with their child. For the five-year-olds, the slide show shows pictures of measuring length, weight, and vaccination, a book used in the dialogue about lifestyle habits, and a picture showing a conversation between a nurse and a parent. As part of their clinical assignment, the nurses were trained to use pictorial support in CHS health visits.

**Fig. 1:**
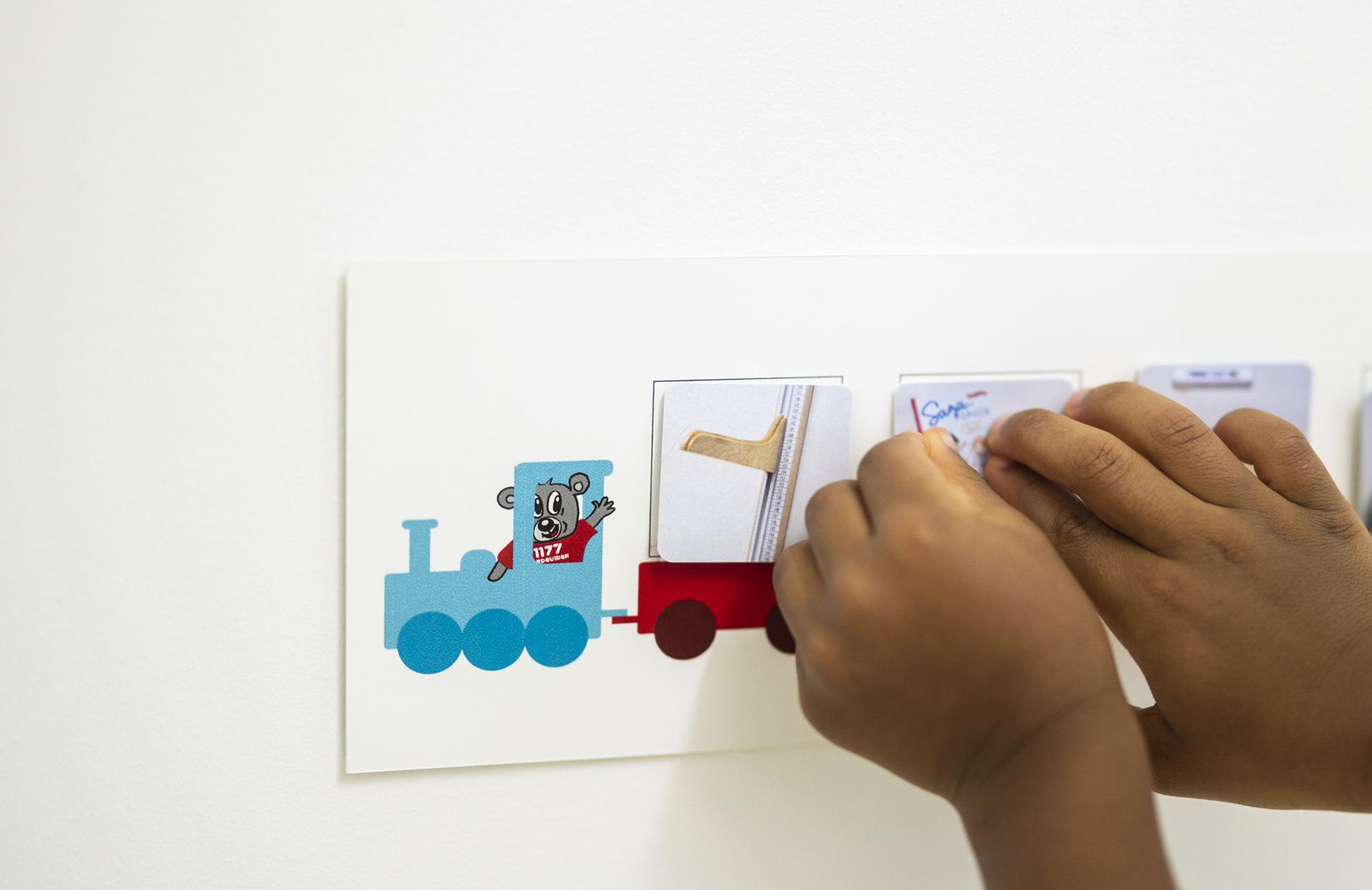
The pictorial support The Train. Photo Johan W Avby

When the child comes to the visit, the pictorial support designed as a train is used (titled The Train; see Fig. 1) to promote the child’s participation. The pictorial support includes the same pictures as on the website. In the health visit the nurse invites the children to choose the picture representing the examination they want to perform and put it on The Train when the examination is completed. Thereby, the child can design the order of the various examinations and have an overview of the different parts of the health visit. The Train is placed either on the table or on the wall in the examination room, (see Fig. 1).

### Participants and inclusion/exclusion criteria

Children and their parents scheduled for their regularly health visit at the CHS at five years of age were asked to participate in the study. Children five years of age were chosen as participants in this study based on their developmental ability to take part in both the health visit for about 30 min and then in the following interview at the same occasion compared to younger children. A prerequisite for being asked was that the child and their parent(s) speak and understand Swedish. A total of 23 children were invited to participate, 17 of whom (11 boys and six girls) participated and six declined to participate. Due to ethical standards, the parents were not asked about the reason for their declined participation in the study.

### Data collection

Data was collected in January and February 2022 via observations of the ongoing health visits and by interviewing the children about their experiences of using pictorial support. The health visits were conducted in the nurses’ ordinary examination rooms at the CHS clinic. The rooms were partly differently designed, but all contained the medical equipment needed to measure the children’s growth, a round table with chairs for the conversation and also a desk with a computer for the nurse’s documentation. Seven different CHS nurses conducted the 17 observed visits, which lasted 25–35 min. The researchers were present during the health visit as a whole. During the observations the researcher (JH or JLJ) sat in the background, making field notes about the child’s actions and expressions in the interaction with the nurse without participating in the visit as naturalistic observation e.g. described by Hammersley& Atkinson [25]. The field notes were written out shortly after the visit to obtain detailed descriptions. The interviews were conducted in a room at the CHC clinic immediately after the visit to facilitate the children’s sharing of their experiences [26, 27]. Further, The Train and the pictures used during the visit were also used during the interview to help the child relate to the visit [28, 29]. The child was encouraged to talk about the visit through open-ended questions, such as *What did you think about using the pictures? Did the nurse listen to what you wanted to say? Did you get to decide anything during the health visit? Is there anything more about the visit you’d like to tell me?* The researcher also used follow-up questions based on the child’s answers. (See supplement 1 for the study specific interview guide.). Each interview took five to ten minutes, and was audio-recorded and transcribed verbatim. One or two of each child’s parents were present during the interview.

### Data analysis

**Fig. 2:**
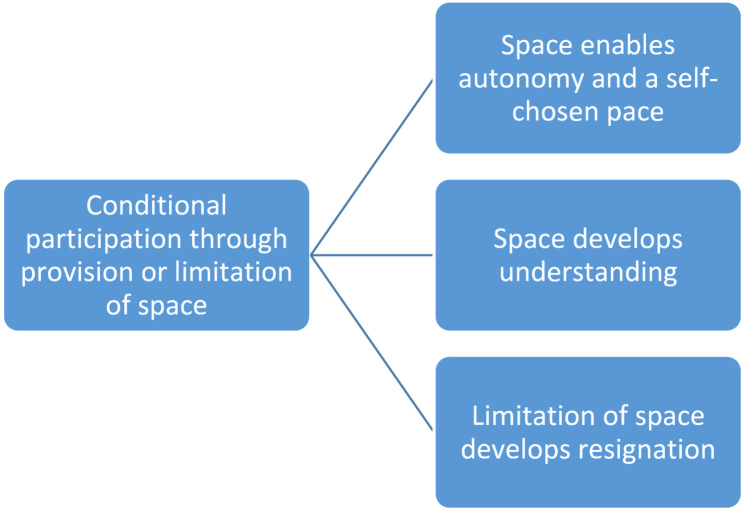
Overview of the results

A hermeneutic approach was used to analyse the observations and interviews [23, 24]. The analysis, performed in collaboration between the first and last authors, began with reading through the field notes from the observation and its accompanying transcribed interview to gain an initial understanding of the child’s experiences of using pictorial support focusing on the child’s opportunity for participation. This was done to grasp the material as a whole [19, 20]. Then, the observation and its accompanying interview were reread, and a decontextualisation-recontextualisation process resulted in an initial abstraction. This process included parts of the transcribed interview being combined with the field notes to form new units of coherent text. In this phase, the authors reflected on the meaning of each unit of text by asking *what* and *how* questions in regard to the text. These questions were based on the purpose and theoretical starting point to gain further understanding: *What are the child’s experiences of the pictorial support? How can the child’s experiences be understood by their actions in relation to the pictorial support and what they say during the interview? How can participation be understood from how they act in relation to the pictorial support and what they express during the interview?* Possible interpretations were constructed as overall understandings showing that the children’s experiences of the pictorial support and participation seemed to be influenced by the child-nurse encounter. The various overall understandings were compared to discover similarities and differences between them. The comparison process made it clear that the children’s experiences of the pictorial support from a participation perspective were conditional on the health visit’s predetermined agenda and how the nurse introduced and guided them using the pictorial support. In this process it was possible to construct an overarching theme, *Conditional participation through provision or limitation of space*. Within this theme, variations are constructed as subthemes: Space enables autonomy and a self-chosen pace; Space develops understanding; and Limitation of space develops resignation (see Fig. 2).

### Ethical consideration

This study was approved by ethical committee, Dnr 2021-06821-01. The children and their parents received written information about the study at the same time they received the health visit notice. The parents provided written consent for the children to participate in the study, and the children gave their oral consent at the health visits.

## Results

### Conditional participation through provision or limitation of space

The children’s participation is conditional due to the content of the health visit, which is predetermined by the National Child Health Programme. It is also conditional due to the nurse’s ability to use the pictorial support in such a way that it promotes their participation rather than simply serving to clarify the visit’s content. The children’s participation occurs within and is shaped by these conditions and these influence their space to enable autonomy and a self-chosen pace or space to develop understanding. The pictorial support becomes a structure for the visit as it contains all the examinations to be performed, and the child has the opportunity to be involved and engaged in the visit on their own terms within this structure. Further, these conditions may also limit the child’s space to be involved and engaged, hampering their opportunity to participate in an already predetermined situation.

### Space enables autonomy and a self-chosen pace

The pictorial support seems to promote the child’s opportunity to be involved and engaged on their own terms. It also helps the child understand the content of the health visit, and facilitates their understanding of why the visit’s various parts are needed. From a participation perspective, the child’s autonomy and self-chosen pace may be described as experiences of being informed, being prepared, taking their time and directing the visit with help from the pictorial support.

In these situations, the child’s participation initially is guided by the nurse. The children are informed about the pictorial support and then encouraged to choose the first picture. The visit then seems to proceed autonomously and at the child’s self-chosen pace as the child chooses a picture, completes the examination and puts the picture on The Train without further encouragement. Another example of self-chosen pace is when a child who has chosen the picture illustrating the talk between nurse and parent puts the picture on The Train to declare an end to the adults’ talk and possibly a desire to be involved. The child may also choose an examination without selecting a picture as they identify the examinations from items in the room, e.g. the scale for measuring weight. They seem to understand how the pictorial support will be used. The children’s willingness to be involved is demonstrated by listening to the nurse’s information as they look at the pictures the nurse shows and by seeking eye contact with the nurse, nodding and showing eagerness. They may also comment on the pictures or The Train, saying for instance, *“recognise them from home*,*” “Mummy has shown”*,* “you understand from the pictures what will happen…and you get to decide the order on The Train”* or *“The Train is fun” (Child 8).*

The children have already been prepared at home, which may give them the prerequisites for being engaged and taking initiative. However, some children who have not been prepared at home still pick up on the procedure and embrace it; they seem to be able to be sufficiently prepared through the information they receive at the visit. Further, they confirm their understanding of the pictures showing the various parts of the visit and why the examinations are needed by nodding in response to a direct question in the interview or by describing the pictures, for example *“look at letters to see if my eyes are good” (Child 13).*

After being informed, a child may take their time before selecting the first picture. The children seem to need to think through what is expected of them, prepare themselves, or consider how to manage the situation. They may also say, *“I chose the first picture because it was fun” (Child 12)*. After this they choose the pictures, and the various parts of the visit are performed more rapidly. Still, a child may slow their pace down to think more before choosing the next picture, e.g. the vaccination, as if needing to think through their management. They may choose the vaccination whenever they’ve decided the right time for it, rather than as the last thing to do. It seems that when they have the opportunity to choose the order of the various parts, this makes it possible for them to be prepared. After the vaccination, the pace of choosing pictures and performing various examinations may increase. Regarding such situations, the children in the interview clarified that they did not have the opportunity to make their own decisions as they could not influence the content of the visit, but that the nurses listened to them. This clarification may be understood as decision-making possibly being related to the things one has chosen, rather than simply the opportunity to choose the order in which predetermined things are to be done. Still, there are also descriptions in which the child says *“First I wanted to pick this [pointing at the scale to measure weight]*,* and then this [pointing at the scale to measure height]*,* and then this [pointing at the talking picture]*,* and then this [pointing at the injection] and this [pointing at the book]” (Child 5).* It appears as if the pictorial support enables children to participate on their own terms.

The children may also use the pictures to direct the visit and regain or maintain the nurse’s attention by bringing up subjects to discuss, for instance pointing at the picture of the vaccination and saying, *“I think it will hurt”*, and then selecting another picture and continuing, *“so I’ll pick the vaccination and then the book” (Child 15)*. These expressions can be understood as the child not yet being prepared to go through with the injection. By planning the order in which they want to perform the remaining examinations, they not only direct the order of examinations through the pictures but may also give themself space to be prepared. Furthermore, the pictures may be used to express feelings, for instance pointing at the picture of the vaccination and saying *“I don’t dare take that” (Child 9)*, and thereafter choosing another picture to give themself a break by managing the visit in another direction. Such actions by the child may give them the space they need to perform the various parts of the visit at their own pace as they eventually manage to perform the whole visit without observable difficulties or resistance. However, there is another nuance here: *In one case a child firmly states a wish to have the injection in his right arm but the nurse wants to give it in his left arm*,* and it is the nurse’s decision that takes priority (Child 6).* The child completes the vaccination without difficulty, but looks worried when he cannot choose which arm to have the injection in.

### Space develops understanding

The pictorial support seems to promote the children’s willingness to be engaged and helps them understand the content of the visit and the procedure. From a participation perspective, the children’s understanding can be described as experiences of being invited to participate, following instructions and taking command.

In these situations, the children are continuously invited to select a new picture, and it seems as if they await further invitations before proceeding, as if trying to figure out how the visit with the pictorial support works. Still, they choose the pictures on their own, look around in the room to identify where the examinations are performed, and perform them without hesitation. They also follow the instructions to put the pictures on The Train. These various pliable actions are understood as the children wanting to use the pictorial support and being willing to be involved and engaged in the various examinations.

After some time of receiving and awaiting instructions, After having received instructions a number of times, the children seem to have understood the procedure and take further command of the situation. They express that they *“chose the picture’*,* which was ‘good” (Child 4)*. Taking command may also include seeking attention when the adults are talking, by picking up the pictures and speaking about those that remain, expressing *“I want to take the injection now”* and *“I want to see the injection; I’m afraid of the injection” (Child 7)*. When the vaccination is completed and the adults continue talking, a child may use the pictures to again demand attention by pointing at them and saying *“there” (Child 7).* The children seem to feel left out when the adults discuss things, as they, in the interviews, give the thumbs up for all the pictures from the pictorial support except the one depicting this. However, they also seem to develop an understanding that they can use the pictorial support to make the visit move in the direction they desire.

### Limitation of space develops resignation

There are also situations where the pictorial support seems to be used solely to clarify the verbal information about the health visit. In such situations, the support is not used to promote the child’s participation or their understanding of why the various parts of the visit are needed. Therefore, the child’s opportunity to be involved and engaged on their own terms is limited, which seems to make them feel resigned in the situation. From a participation perspective, space limitation may be described as children’s experiences of being restrained and kept ignorant.

It can be noted that a child’s space is limited when they tell the nurse they recognise the pictures, say they know how The Train works, and listen and confirm the nurse’s information by nodding but still have no space to influence the order in which the various examinations are performed. Their opportunity to engage on their own terms seems to be restrained by the nurse’s predetermined idea about the best order in which to perform the examinations, as the children are not allowed to select a picture on their own. The children confirm this in the interviews by expressing that they *“did not have the opportunity to choose the pictures” (Child 14)* independently. They seem to resign themselves to refraining from engaging in the situation on their own terms and accept the given conditions. Still, they act in a pliable way in relation to the nurses’ instructions, perform the various examinations, and put the pictures on The Train after each examination. However, they try to create some space for themselves by performing the examinations at their own pace. They may also use the pictures to get attention from both their parents and the nurse by waving at them. On such occasions the children may need more support from their parents, as shown by their seeking bodily contact with them.

Further, a child’s space can be regarded as limited when they try to use the pictures to express worry about certain examinations. This occurred, for instance, in relation to the vaccination: While the children is provided with information about the pictures and The Train, they repeatedly tryto say *“don’t want the injection*” and *“don’t want the injection*,* want to go home’*” (Child 17). Still, the nurse urges the child to proceed with the visit by giving them a picture, encouraging them to undergo the examination, and putting the picture on The Train while ignoring the child’s concern and need to be listened to. The child acts pliably and performs the examinations, but the vaccination must be performed using restraint. After this, the child does not move any more pictures to The Train, which may be understood as resigning themself to relinquishing their involvement and engagement in the situation.

## Discussion

The findings show that children’s experiences of using pictorial support, as in this study The Train, from a participation perspective is conditional by the nurses, as their guidance may provide the children with *space to enable autonomy and a self-chosen pace*, or *space to develop understanding*. The nurse’s guidance may also contribute to *limitation of space which develops resignation*.

In the discussion, we try to illuminate the findings from a theoretical perspective by using Davies et al. [30] framework for listening to children in health care encounters, which is based on Lundy’s [31] conceptualisation of Article 12 [11]. Davies et al. [30] use the concepts of space, voice, audience and influence. These concepts are one way to concretise further how children can contribute to a health care situation [16], i.e., participate from their perspective.

*Space* means the children’s opportunity to express their views [31], and Davies et al. [30] state that space is crucial for recognising what is important to children [25]. In this study, space is provided through the pictorial support as preparation and a tool for accomplishing the health visit. Further, it seems that space is crucial, as participation in the visit is conditioned by how the children’s space is provided or limited as confirmed by several studies [4, 32–34]. Harder et al. [32] shows in a study with five-year-old children that children perceive themselves as actors in the encounter but that their opportunity to take part may be compromised. Children’s experiences of less space may contribute to perceptions that the health care encounter is difficult. Further, Lundberg et al. [34] describe how children’s participation is enabled by physical and mental space From the present and previous studies, it is possible to suggest that nurses’ actions in health care encounters with children may take advantage of the pictorial support as it allows the children to be active and have some control in the situation. The children can act at their own pace by directing what will happen next, by how they choose the pictures.

In this study, space refers to a psychosocial space in which the children can reflect on, take in and understand the situation and express their views. Davies et al. [35] confirm our finding in an empirical study with children in hospital. In that study, the concept of space is broadened, and is described as support, encouragement, comfort and trust. Providing space means addressing the children directly rather than talking over their head to their parents [30]. The pictorial support is a way to facilitate the communication between child and nurse. They choose the pace by interrupting the conversation between the nurse and their parents by putting the picture of the adults’ talk on The Train to show that the conversation is over. Therefore, the pictorial support provides the child with an opportunity to practice their right to have an impact in the situation. A child’s actions to make use of their rights to participate can depend on how their actions are encountered. Therefore, an encounter may end up in mutuality in which the child and the nurse cooperate in joint attention or alienation in which the child turns away from the nurse or the child is ignored by the adults [36] depending on how the nurse decides to make use of the pictorial support. Furthermore, Davies et al. [30] argue that there is a risk involved with tools aimed at providing space if they are used as tick boxes. This is also illustrated in the present study when the child is given pictures by the nurse based on her own predetermined agenda. It becomes obvious that simply using the pictorial support is not in itself a way of providing space; it also must be used wisely to act in the child’s best interests and promote their participation. The support is not used wisely when it creates a limitation of space, with the consequence that the children resign themselves and are not able to be engaged even though they understand how the pictorial support works. In summary, the theoretical concept of space, which appears to be a supporting pillar in children’s participation, can be implemented in an actual health visit by using pictorial support wisely.

*Voice* refers to the children’s right to express their view [16], and involves receiving adjusted information and time for understanding [31]. The pictorial support can be understood as a tool for implementing the children’s rights through such adjustments. The children described how the pictorial support helped them understand the content of the visit. Pictorial support as a way to understand the content of a health visit is also confirmed by the children in other studies [7, 37]. Further, the children describe the Train as fun as they could decide the order of the examinations on their own, so they were able to act autonomously. This is in line with Stålberg et al.‘s [38]. study in which the children describe themselves as essential actors having the possibility to participate on their terms. Children themselves expressed voice as having the opportunity to have a say and speak up and being allowed to use their voice to talk about both agreements and disagreements [35]. Further, pictorial support is valuable as an aid in preparing children for health care encounters, and provides a sense of security and control [1, 22]. It may facilitate child-centred care in which the child’s voice comes first [39] and the child is viewed as the key person in the situation [20] in the triad partnership of child, parent and nurse. In summary, the present study shows that pictorial support allows the child to be engaged and facilitates bringing up subjects to discuss by pointing at a picture. The pictorial support aid them in expressing their voice and maintaining the nurse’s attention in the encounter, but they depend on the nurse’s willingness to listen actively to the children’s voices.

*Audience* involves the child’s views being listened to and considered [31]. In this study, audience is shown when the nurse responds to the child by listening to them and showing them interest in how they choose the pictures. When nurses interact with children by listening and talking to them and having a friendly facial expression, guidance through the situation is facilitated [40, 41]. Further, nurses alternate between flexible and directive strategies to encourage and guide the children through the various examinations of a health visit to support them [4, 18]. This promotes the child’s autonomy, and they become engaged in the encounter [30].

For children, encounters in health care often imply encounter’ with unknown adults, which children describe as causing difficulties in daring to talk [8]. To not recognise the children’s expressions, increase the risk of the nurse controlling the health visit through directed strategies without acknowledging the child’s perspective [4, 18]. Lack of audience is shown in the present study when the nurse ignores the children’s attempts to tell them that they understand the pictorial support and the nurse instead follows her own predetermined idea of how to accomplish the visit. When the results of the present study are compared with the results of Harder et al. [42], nurses still tend to be primarily guided by the tasks in the national health program instead of catering for each child’s needs. This shows a lack of the skills necessary to consider children’s views [30] and a need to develop actions to promote children’s opportunities to be engaged [43].

These variations of children’s rights to have their opinions listened to and considered illustrate that, although pictorial support can help children express their opinions, it depends on how the nurse acts as an audience. As children at age five may still prefer communicating through nonverbal means, the nurse must consider and recognise verbal and nonverbal communication. Pictorial support can make it easier for the child to tell, but nurses must respond to the child’s expressions and thereby act as an active audience.

*Influence* concerns how the health care professionals [30, 31] act upon children’s views. The pictorial support seems to promote the children’s opportunity to influence the situation on their own terms by choosing the pictures independently and determining the next step of *the visi*t. It seems like the children want to participate by influencing the health visit from their own perspective which is consistent with previous studies. Harder et al. [42] found that children as young as four years of age negotiate by using affirmative and delaying strategies to influence the health visit. Furthermore, Kleye et al. [12] found that children want to choose their own strategies to manage fear and pain in health care situations. The opportunity to make choices and having options are described as elements in having influence [35], as well as understanding the purpose of the various parts in a health visit [7]. In our study the children seem to use the pictorial support to make the visit move in their desired direction. However, they still seem to need some help in the situation, as they await the nurse’s instructions before choosing a new picture. The nurses act upon the children’s views by instructing them until they understand the procedure and can choose the pictures independently. On the other hand, in the interviews the children say that they understand that they can influence the order but not the content. This is in line with Harder et al. [32] who show how five-year-old children depict themselves as being actors of attention but at the same time they show their less opportunity to influence the situation [32]. Also, Lundberg et al. (34) and Davies et al. [35] confirm children’s less influence as the children in their studies express that their actual influence is small and is rather based on the professionals’ perspective on what influence is for children. All visits in the Swedish CHS have a predetermined content with the purpose to promote the child’s health and development. Therefore, the nurse’s actions towards the child and her skills in clarifying this condition will affect the child’s experiences of real influence [4]. We stress in line with Carlsson et al. [37] that the pictorial support may increase the children’s understanding and provide them with comfort. In this study, a lack of influence is shown when the nurses limit children’s opportunity to be engaged as the nurses themselves choose the pictures based on their predetermined idea of the best order, or when they give the vaccination under restraint. In such cases resignation develops, and the child does not show any attempt to be engaged. The child’s opportunity to be involved and engaged on its own terms is limited [25]. In encounters such as this, the child is left with a sense of alienation instead of mutuality, which hampers their opportunity for participation [36]. The pictorial support will help the children to participate from their own perspective. Still, they are dependent on responsible adults, nurses as well as parents, paying attention to and acting on the children’s actions rather than from their own grown-up perspective.

### Methodological considerations

One strength is that the observations and interviews were used as complementing data collection methods to make it possible to grasp the children’s experiences of using pictorial support (2729). The observations were needed to add context to the interviews and vice versa to provide understanding of the children’s perspective [27, 29]. While the hermeneutic analysis contributes one possible description of the children’s experiences of using pictorial support, there is an understanding among the authors that there may be other reasonable descriptions.

## Conclusion

In summary, children’s experiences of using pictorial support from a participation perspective seem to be conditional by circumstances in the situation. It is not enough to provide space and let children use their voice through pictorial support to promote their participation. The children have to have an audience willing to listen or opportunities to influence the visit. Nurses encountering children need skills to promote children’s participation and need to reflect on whether their guidance is promoting a child’s participation or whether they are just too focused on carrying out a procedure [44]. By departing from a framework that promotes listening to children in health care encounters, nurses could strengthen their ability to reflect on how best to support children in expressing their views.

Further research and development on how pictorial support can promote participation during health visits with young children in CHS needs to include experiences from younger children, parents, nurses, and physicians.

## Supplementary Information

Below is the link to the electronic supplementary material.

Supplementary Material 1

## Data Availability

The data that support the findings of this study are available on request from the corresponding author. The data are not publicly available due to privacy or ethical restrictions.

## Abbreviation

CHS: Child Health Services
