# Supplementary material for: Pictorial support in health visits in Child Health Services provides or limits children’s space for participation
Source: BMC Health Serv Res. 2026 Jul 3;26:910. doi: 10.1186/s12913-026-15049-1 (PMC13330147; doi:10.1186/s12913-026-15049-1)
Supplement: Supplementary file 1 — Supplementary Material 1 [file 12913_2026_15049_MOESM1_ESM.docx]

Interviewguide

The Train and the pictures used during the health visit were used during the interview to help the child relate to the health visit.

Now that you've been on your health visit, I'm a little curious about how you thought it was, so I have some questions.

Have you seen the pictures before coming here for the health visit?

What did you think about using the pictures during the health visit?

Which image do you want to start talking about?

Follow up on what was good or less good and why?

What do you think other children think about using these pictures?

Is there anything else about the pictures you would like to tell me?

When you were with the nurse today, did the nurse listen to what you wanted to say?

Follow up on what was good or less good.

Did you get to decide anything during the health visit?

Follow up on what was good or less good.

Is there anything more you want to tell me about the health visit?
